# Concurrent habitat and life history influences on effective/census population size ratios in stream-dwelling trout

**DOI:** 10.1002/ece3.196

**Published:** 2012-03

**Authors:** Sebastian Belmar-Lucero, Jacquelyn L A Wood, Sherylyne Scott, Andrew B Harbicht, Jeffrey A Hutchings, Dylan J Fraser

**Affiliations:** 1 Department of Biology, Concordia University7141 Sherbrooke St. West, Montreal, QC, Canada, H4B 1R6; 2 Department of Biology, Dalhousie University1455 Oxford St, Halifax, NS, Canada, B3H 4J1

**Keywords:** Abundance, effective population size, habitat, life history, trout

## Abstract

Lower effective sizes (N_e_) than census sizes (N) are routinely documented in natural populations, but knowledge of how multiple factors interact to lower N_e_/N ratios is often limited. We show how combined habitat and life-history influences drive a 2.4- to 6.1-fold difference in N_e_/N ratios between two pristine brook trout (*Salvelinus fontinalis*) populations occupying streams separated by only 750 m. Local habitat features, particularly drainage area and stream depth, govern trout biomass produced in each stream. They also generate higher trout densities in the shallower stream by favoring smaller body size and earlier age-at-maturity. The combination of higher densities and reduced breeding site availability in the shallower stream likely leads to more competition among breeding trout, which results in greater variance in individual reproductive success and a greater reduction in N_e_ relative to N. A similar disparity between juvenile or adult densities and breeding habitat availability is reported for other species and hence may also result in divergent N_e_/N ratios elsewhere. These divergent N_e_/N ratios between adjacent populations are also an instructive reminder for species conservation programs that genetic and demographic parameters may differ dramatically within species.

## Introduction

Habitat features profoundly shape the demography and life history of populations by affecting, for example, population abundance, age- and size-at-maturity, and intraspecific competition (Roff 1992; Krebs 1999; Ylikarjula et al. 1999). Demography and life history, in turn, can have important consequences for the amount of genetic variability within populations. Most populations, notably, have census sizes (N) that are larger than their effective population sizes (N_e_), a parameter governing the rate of loss of genetic diversity through random genetic drift in a finite-sized population (Frankham et al. 2002). Effectively, N_e_ represents how many and to what extent individuals in a population contribute to the gene pool from which the next generation will be drawn (Wright 1931).

Several ecological factors can reduce the ratio between N_e_ and N (N_e_/N), such as fluctuations in N, variance in individual reproductive success, and unequal sex ratios (Frankham et al. 2002; Palstra and Ruzzante 2008; Lee et al. 2011). These factors can result in a lack of relationship between N_e_ and N, a key point for conservation practices that aim to balance demographic and genetic concerns (Ardren and Kapuscinski 2003; Fraser et al. 2007). Often however, the potential for ecological factors to have interacting effects in reducing N_e_/N is poorly understood, in part because such factors are not considered concurrently with the habitat features that drive them. This information gap represents the impetus for the present study on stream-dwelling populations of a salmonid fish, the brook trout (*Salvelinus fontinalis*).

Aspects of the habitat and mating system of brook trout might result in different N_e_/N ratios among populations. For instance, specific and sometimes limited habitat requirements for the successful rearing of fertilized eggs, such as groundwater seepage, can put a premium on the availability of spawning sites (Blanchfield and Ridgway 1997, 2005; Guillemette et al. 2011). Indeed, competition for access to spawning sites between females and for access to females between males, lead to increased variance in reproductive success among members of both sexes (Blanchfield et al. 2003; Blanchfield and Ridgway 2005; Theriault et al. 2007; Kanno et al. 2010). Furthermore, brook trout populations exhibit considerable life history and phenotypic differentiation in relation to local habitat features, including dramatic body size and morphological differences (Hutchings 1996; Fraser and Bernatchez 2005). These might indirectly affect intraspecific competition for spawning resources, and hence the relationship between N_e_ and N through, for example, density-dependent effects.

Cape Race, Newfoundland, Canada (bounded by 53°16′W, 46°45′N, 53°04′E, 46°38′S; Hutchings 1990) harbours many small, resident, stream populations of brook trout. These pristine and near-pristine populations are considered to be isolated since at least the late Wisconsinan glaciation (Rogerson 1981; see also Ferguson et al. 1991). In fact, most Cape Race streams terminate off 30–50 m cliffs into the Atlantic Ocean, including those in this study. Positioned at a fine geographic scale (15 km × 10 km), the populations experience the same climate but exhibit diverse life histories (Hutchings 1990, 1993, 1994, 1996). They are also subjected to negligible fishing pressure due to the small body size of the trout (typically 100–150 mm; Hutchings 1990, 1993). These populations are thus useful for assessing the concurrent roles of habitat features and life history in driving N_e_/N.

**Figure 1:**
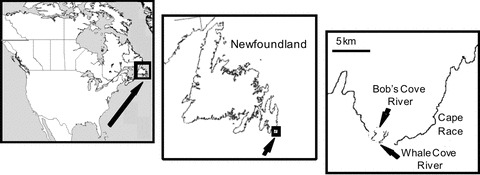
The geographic locations of Bob's Cove and Whale Cove rivers on Cape Race, Newfoundland, and in relation to the rest of North America. Figure 1.

Two Cape Race streams separated by only 750 m, Bob's Cove and Whale Cove Rivers, are of particular interest (hereafter BC and WC, respectively; Fig. 1). An initial assessment of the two streams showed some notable habitat differences between them. Namely, despite having a larger drainage area occupied by trout, BC has fewer spawning sites relative to WC. BC is also shallower, faster in stream velocity, and appears to vary more interannually in summer temperature than WC (Table 1). The spawning sites in both streams are characterized by having groundwater seepage (Appendix 1).

**Table 1:** Summary of brook trout habitat data collected in 2010 and 2011 for Bob's Cove river and Whale Cove river in southeastern Newfoundland.

	Bob's Cove river		Whale Cove river	
Habitat variable	2010	2011	2010	2011
Mean pH (± 1 SE)	6.41 (0.14)	6.40 (0.03)	6.65 (0.21)	6.87 (0.24)
Mean temperature (°C, ± 1 SE)^1,2^	9.77 (0.18)	17.52 (0.52)^3^	11.88 (0.76)	12.14 (0.32)
Mean velocity (m/s, ± 1 SE)^1,2^	0.20 (0.05)	0.23 (0.05)	0.10 (0.02)	0.09 (0.03)
Proportion riparian cover (± 1 SE)^1^	0.01 (0.01)	0.08 (0.02)^3^	0.08 (0.02)	0.09 (0.02)
Number of plant species/transect (± 1 SE)^2^	1.30 (0.16)	2.70 (0.32)^3^	1.39 (0.22)	1.39 (0.22)
Proportion of transect with vegetation (± 1 SE)	0.34 (0.08)	0.52 (0.10)	0.41 (0.09)	0.41 (0.11)
Bank width (cm, ± 1 SE)	217.71 (24.95)	201.62 (27.01)	224.22 (48.83)	183.56 (42.02)
Mean depth (cm, ± 1 SE)^1,2^	16.57 (1.27)	14.12 (1.06)^3^	20.00 (2.07)	18.20 (1.53)
Stream length (km)	2.05		1.82	
Drainage area (km^2^)	1.41		0.92	
Drainage area occupied by trout (km^2^)	1.41		0.37	
Invertebrate abundance (mean, range)	1536.3 (695–2814)	4376.7 (730–8400)	1457.5 (288–2720)	3033.3 (1910–4940)
Spawning sites	1	1	5	6
Spawning site area (m^2^)	31.38	31.38	45.28	47.73

1,2 Significant differences in the means between the two streams in 2010 and 2011, respectively, based on analyses of variance (ANOVA).

3 Temporally significant differences in the means between years within streams, based on ANOVA.

With respect to the habitat differences in stream depth and spawning site availability, we can formulate two hypotheses regarding the concurrent influence of habitat and life history on the relationship between N_e_ and N in both populations. First, in fishes, selection tends to favor smaller body size and/or earlier age-at-maturity in shallower streams because larger fish have reduced overwintering survival and/or higher predation risk than smaller fish due to a lack of deep habitat (Miller 1979; Hutchings 1990, 1993; Gende et al. 2001). We might therefore expect BC fish to be smaller than WC fish. Second, lower spawning site availability may generate greater competition among spawning trout in BC than in WC (see Blanchfield and Ridgway 2005). We might therefore expect N_e_/N ratios to be lower in BC than in WC, especially if fish densities are higher in BC due to smaller body size associated with shallower depth that should exacerbate spawning competition. And yet shallower, faster streams can also be more sensitive to environmental changes (Allan and Castillo 2009); this may be the case for BC as suggested by interannual summer temperature data. Perhaps lower N_e_/N in BC might be additionally expected because greater environmental variability could generate more fluctuations in N, and a potential downward reduction in N_e_ (Waples et al. 2010).

We tested our hypotheses by combining our initial habitat assessment with a comparison of size- and age-at-maturity and N_e_/N ratios between the two streams. These analyses were supplemented with a comparison of sex ratios and breeding female characteristics (egg sizes and fecundity) to consider other ecological factors that might affect N_e_/N ratios within our study populations.

## Materials and Methods

### Habitat data

The discharges of BC and WC are situated at 53°12.946’W, 46°38.219’N, and 53°12.307’W, 46°38.051’N, respectively (Fig. 1). The following habitat data were collected from BC (21 100-m transects, denoted with GPS, global positioning system, coordinates to within 3 m) and WC (18 similar GPS recorded 100-m transects) in the summer of 2010 and 2011: pH, temperature, stream velocity, proportion of riparian cover, number of plant species per transect, proportion of vegetation, bank width, and stream depth. We furthermore quantified stream length and drainage area, as well as invertebrate abundance in each stream using kick-and-drift sampling at three randomly drawn locations. Invertebrate abundance was based on total individual counts across taxa. Finally, the total number of spawning sites and their approximate area was recorded for each stream in the fall of 2010 and 2011. Spawning sites were easily recognizable by dense aggregations of sexually mature trout, excavated redds, and by the presence of *Montia fontana*, a herbaceous annual plant that is indicative of groundwater seepage at Cape Race. Details of the quantification of different habitat characteristics and their relevance to brook trout survival and growth are found in Appendix 1.

### Life-history data

#### Sex ratios, age- and size-at-maturity, female fecundity, and egg size

In the fall of 2009 and 2010, we estimated adult sex ratios in each stream based on individuals that could be sexed confidently from electrofishing surveys; total length and age of a subset of mature individuals were also determined and compared between streams. Age was assessed from standard scale analysis and defined as the number of completed winter seasons, for example, 1+, 2+, 3+. Finally, we collected eggs from mature females (BC, n = 12; WC, n = 21) in the fall of 2010 to record and compare fecundity and egg size. Egg size was estimated by measuring the diameter of 10 randomly selected eggs per female, using a known size standard.

### Genetic and demographic data

#### Genetic sampling

Tissue samples of individuals from both populations were obtained as adipose fin clips (BC = 158; WC = 139) during the fall of 2009. To obtain a randomly sampled mixed-cohort representation in each stream (ages: 1+ to 4+ in BC and 1+ to 5+ in WC), fish were captured using 3-min electrofisher surveys conducted at each 100-min interval from the stream mouth.

Tissue samples were stored in 95% ethanol until DNA was extracted using a modified phenol–chloroform protocol. Microsatellite polymorphism was analysed at six loci using fluorescently labelled primers (triplex: SfoC28, SfoC113, SfoD100; SfoD91, T. L. King, US Geological Survey, unpublished; duplex: Sco220, DeHaan and Ardren 2005; and Ssa408uos, Cairney et al. 2000). PCR amplification of the loci was performed in a total of 10-µL reaction volume, containing 1µL of 10X TSG buffer, 1 µL of 20-mm MgSO_4_, 1 µL of 2-mm dNTPs, 0.3 µL of each of 10-mm forward/reverse primers, 0.1 µL of TSG polymerase, 4.6–4.8 µL of ddH_2_O, and 2µL of genomic DNA. The PCR conditions were an initial denaturation at 94°C for 3 min, followed by 30 cycles of denaturation for 30 sec at 94°C, annealing at 57°C for 30 sec and elongation at 72°C for 1 min, and a final elongation at 72°C for 15 min. Amplified fragments were separated electrophoretically in an 6% polyacrylamide gel using the LICOR 4200 global IR2 system, and allele sizes were scored based on a fluorescently labeled size standard.

#### Within-sample genetic diversity and population differentiation

We used GENEPOP 4.0 (Raymond and Rousset 1995) to quantify alleles per locus and observed and expected heterozygosities, to verify Hardy–Weinberg equilibrium (HWE) expectations of genotypic frequencies (across all loci in each stream and at each locus), and to test for genotypic disequilibrium between all loci pairs. In order to confirm the assumption that populations from both streams represented genetically independent units, we calculated Weir and Cockerham's (1984)θ_ST_ using fstat 2.9.3. Additionally, since population subdivision is known to affect N_e_ estimates, structure 2.1 (Pritchard et al. 2000) was used to evaluate the genetic structure of both populations by modelling the likelihood of K = 1–5 subpopulations per stream.

#### Contemporary effective population sizes

We used two approaches to estimate contemporary N_e_ of the mixed-cohort sample for both BC and WC, as different approaches for estimating N_e_ have varying assumptions and assume discrete generations while our iteroparous study species has overlapping generations. An implicit assumption underlying our analyses, therefore (and one without testing in the literature thus far), is that N_e_ estimates based on our mixed-cohort samples should roughly correspond to N_e_ of a generation (Waples and Do 2010). First, N_e_ was calculated using the approximate Bayesian computation method implemented in onesamp (Tallmon et al. 2008). onesamp uses summary statistics from the data input by the user, and calculates N_e_ for 50,000 simulated populations, accepting N_e_ values for populations with summary statistics close to those obtained from the input data (Tallmon et al. 2008). We used the priors N_e_ max = 5,000 and N_e_ max = 1,000 (N_e_ min = 50) for BC and WC, respectively, because estimates of N reached these values in the two streams (see Results). Second, N_e_ was calculated using the linkage disequilibrium method implemented in LDNe (Waples and Do 2008). The principle behind this approach is that linkage disequilibrium (nonrandom associations among alleles at different loci) should increase as N_e_ decreases (i.e., as genetic drift increases) (Waples and Do 2008). For LDNe, we excluded alleles only with frequencies of <0.02 to increase precision without generating too much bias in our N_e_ estimates given the modest number of loci (six) but the large sample sizes used to estimate them (*n*= 158, 139) (see Waples and Do 2010).

#### Census population sizes

We used either the Schnabel (1938) or Petersen (1896) method to estimate adult census population sizes (N) in both streams between July and October in 2010 and 2011. We calculated N as breeding adults only. Breeding adults were easily distinguished from nonbreeding adults in September/October by the presence of sperm or ripe (or nearly ripe) eggs following a gentle stroking of the abdominal cavity. Multiple recapture events (WC, four; BC, two) were performed in 2010 (Schnabel method applied); a single recapture event was carried out in each stream in 2011 (Petersen method applied).

In July 2010, we tagged 120 (BC) and 133 (WC) adult fish (ranging from 100–189 mm) across all reaches of each stream; similar tagging was carried out in July 2011 (BC, *n*= 246; WC, *n*= 150). Individuals were inserted with FD-68B Fine Fabric Anchor Tags (Floy Tag and Manufacturing Inc. Seattle, Washington, USA). In late September of each year, we then recovered tagged individuals in each stream based on an electrofishing survey of the entire stream; in 2010, we also simultaneously tagged more individuals (BC: *n*= 78; WC: *n*= 91). Subsequently, a third electrofishing survey was performed on both streams in early October 2010 with additional tagging (*n*= 43) in WC only; fourth and fifth parallel surveys (without tagging) were also repeated in WC in mid October. A regression plot of the proportion of tagged trout on the number of previously marked in WC in 2010 was linear, suggesting that the assumptions of the Schnabel method had been met (N was constant across recapture events; sampling was random; individuals had equal recapture probabilities). In 2011, we did not recover sufficient numbers of individuals tagged in 2010 to assess other relevant parameters such as age-specific survival (i.e., BC, *n*= 1; WC, *n*= 18).

#### Effective-census population size ratios

A caveat of our research is that our mixed-cohort N_e_ estimates (based on genetic samples collected in 2009) predominantly correspond to N for that stream in the years 2004–2007 (i.e., the parents of the mixed-cohort sample), not the years 2010 and 2011 for which our N estimates were calculated. For two principle reasons, however, we feel that our 2010/2011 N estimates are reasonable proxies for comparing N_e_/N ratios between BC and WC. First, the geographic scale at which N_e_/N ratios are being compared is very small. At the very least, the populations thus experience similar climatic fluctuations that in turn may dampen any differential fluctuations in abundance across both streams. Second, our N data, albeit from only two consecutive years, suggest that BC and WC fluctuate proportionally in breeding adult abundance (see Results).

## Results

### Habitat characteristics

Based on assessed habitat features, BC was significantly shallower (0.25 times) and faster in velocity (2.26 times) than WC in both sampled years; BC was also 2.1°C colder than WC in the summer of 2010 yet 5.4°C warmer in 2011 (Table 1). BC also contained fewer spawning sites and less spawning area than WC despite having a larger drainage area occupied by trout (Table 1). Finally, BC had more riparian cover than WC in 2010 but not in 2011; this unexpected difference most likely reflected the characteristics of riparian cover at BC and WC. For example, riparian cover at these two streams consists entirely of grass and sedge species that might vary more widely with environmental conditions between years simply due to structural differences compared with tree cover. Alternatively, the difference may have been due to fine-scale GPS imprecision (i.e., resolution was only within 3 m; Table 1).

### Life-history characteristics

Sex ratios of mature adult trout did not vary between the two streams (BC: 82♀ vs. 90♂; WC: 101♀ vs. 99♂; χ^2^= 0.19, df = 1). However, BC fish were smaller and matured earlier than WC fish (123.4 ± 2.1 mm vs. 137.9 ± 1.8 mm, Wilcoxon rank-sum test, U = 3749, P < 0.001; 2.7 ± 0.1 vs. 3.2 ± 0.1 years, U = 4252, P < 0.001; Table 2); these differences held for each sex when analyzed separately and were greater for females (all P < 0.01, data not shown). BC females also had smaller eggs than WC females (Student's *t*-test, t =–5.145, P < 0.001), but there was no significant difference in fecundities between the streams (t =–1.493; P = 0.146) (Table 2). Variances in body size, age-at-maturity, egg size, and fecundity also did not differ significantly between the streams (F-ratio tests, all F = 0.02–0.743, P = 0.12–0.92).

**Table 2:** Summary of age-specific lengths for sexually mature brook trout in Bob's Cove river and Whale Cove river in southeastern Newfoundland, across 2009 and 2010, as well as female fecundity and egg size data for 2010 (averages across age classes reported; n = 12 for Bob's Cove river and n = 21 for Whale Cove river).

Population	Females				Males	
	Age (n)	Length^1^ (mm)	Fecundity	Egg size (mm)	Age (n)	Length^1^ (mm)
Bob's Cove river	1+ (2)	81.5 (67, 96)			1+ (3)	100.7 ± 1.9
	2+ (21)	108.6 ± 1.4			2+ (12)	110.8 ± 1.5
	3+ (22)	133.1 ± 1.1	54 ± 9	4.1 ± 0.1	3+ (4)	128.3 ± 2.8
	4+ (10)	146.0 ± 1.2			4+ (5)	153.8 ± 6.9
Whale Cove river	1+ (0)				1+ (6)	104.3 ± 1.0
	2+ (14)	116.1 ± 1.6			2+ (21)	114.1 ± 1.1
	3+ (32)	134.2 ± 1.2	79 ± 12	4.7 ± 0.1	3+ (19)	131.5 ± 1.2
	4+ (21)	155.2 ± 2.2			4+ (26)	151.2 ± 1.4
	5+ (5)	191.4 ± 9.5			5+ (7)	176.9 ± 3.6

1 1 SE, n = sample size.

### Genetic and demographic characteristics

#### Within-population genetic variability

No exact tests for genotypic linkage equilibrium were significant, suggesting independence of the six loci used. Furthermore, no deviations from HWE were detected across loci within streams, as well as at each locus across both streams (all P-values > 0.05). The six loci were moderately polymorphic (3–11 alleles/locus), moderately heterozygotic (range: 0.36–0.84), and exhibited similar mean characteristics across the two streams (Table 3). “Private” alleles were detected at all six loci and were numerically similar in the two streams (BC, *n*= 8; WC, *n =* 10), but all of these alleles have been found in other, adjacent Cape Race streams (Morrissey 2009). We confirmed that BC and WC were genetically distinct (θ_st_= 0.182, 95% confidence intervals (CI) = 0.090–0.285) and contained no subpopulation structure (i.e., clustering models assuming K = 1 subpopulation were strongly supported over K = 2, 3, 4, or 5 in each stream; details in Appendix 2).

**Table 3:** Genetic diversity characteristics within Bob's Cove river and Whale Cove river brook trout populations in southeastern Newfoundland, based on six microsatellite loci.

	Bob's Cove river		Whale Cove river	
Locus	A	H_o_	A	H_o_
SfoC28	3	0.36	3	0.41
SfoC113	6	0.63	5	0.73
SfoD91	7	0.59	6	0.76
SfoD100	4	0.57	5	0.59
Ssa407	8	0.72	11	0.84
Ssa408uos	5	0.75	5	0.54
Multi-locus means	5.3	0.60	5.8	0.65

A = allelic richness; H_o_= observed heterozygosity.

#### Effective/census population size ratios

Point estimates of N_e_ using onesamp were similar between the two streams and had highly overlapping 95% CI (Table 4). The point estimate of N_e_ of BC using ldne was 2.43 times greater than the WC estimate, with upper 95% CI including infinity (Table 4). Conversely, the BC estimates of N were 6.94 and 6.37 times larger than WC (for 2010 and 2011). Across years, the 95% CI for N estimates did not overlap between streams, but they overlapped within streams, and point estimates of N fluctuated proportionally in the two streams (Table 4). Collectively, point estimates of N_e_/N ratios were 2.39–6.05 times smaller in BC relative to WC (Table 4). These overall results did not change markedly when excluding rare alleles with frequencies of <0.01 as opposed to <0.02 using LDNe (see Waples and Do 2010).

**Table 4:** Point estimate N_e_/N ratios within brook trout populations occupying Bob's Cove river and Whale Cove river in southeastern Newfoundland.

	Bob's Cove river	Whale Cove river	*N_e_*/*N* (Whale Cove) divided by *N_e_*/*N* (Bob's Cove)
1*N_e_* (95% CI)	170 (116–324)	146 (127–201)	
2*N_e_* (95% CI)	355 (110–∞)	146 (69–383)	
*N* (95% CI), 2010	5584 (3992–8392)	805 (627–1126)	
*N* (95% CI), 2011	3379 (3024–3856)	582 (496–716)	
With 2010 *N* data			
1*N_e_*/*N* point estimate	170/5584 = 0.030	146/805 = 0.181	6.05
2*N_e_*/*N* point estimate	355/5584 = 0.064	146/805 = 0.181	2.85
With 2011 *N* data			
1*N_e_*/*N* point estimate	170/3379 = 0.050	146/582 = 0.251	4.99
2*N_e_*/*N* point estimate	355/3379 = 0.105	146/582 = 0.251	2.39

CI = confidence intervals.

1 *N_e_*=onesamp.

2 *N_e_*=ldne.

## Discussion

Our results suggest a plausible mechanism for how the interaction between ecological and evolutionary factors can produce disparate N_e_/N ratios between adjacent populations at fine geographic scales. Local habitat features govern the biomass of trout produced on a per area basis in each stream. Yet they also indirectly elicit higher trout densities in BC by favoring smaller body size and earlier age-at-maturity; the combination of higher densities and lower breeding site availability in this stream subsequently results in more competition among breeding trout (irrespective of the exact cause of earlier age-at-maturity). Ultimately, the resulting increased variance in individual reproductive success leads to a greater reduction in N_e_/N relative to WC.

Mean invertebrate density was higher in 2011 for both study streams, however comparisons between study streams suggested no significant differences in the productivity of their habitats. Assuming then that the drainage area occupied by trout is a reasonable predictor of trout biomass (Allan and Castillo 2009), BC should have a total of 3.81 times more trout than WC (Table 1). When additionally accounting for the average body size difference between BC and WC adult trout from length–mass regressions in each stream (1.49 BC trout = one WC trout; data not shown), BC is expected to have 5.68 times more trout than WC. This combined estimate is quite consistent with our mark-and-recapture estimates of 6.94 and 6.37 times greater N in BC than WC, for 2010 and 2011, respectively. Although an explanation for why N_e_/N ratios were 2.4–6.1 times lower in BC than in WC is perhaps less clear, the higher proportional trout density in BC coupled with smaller total spawning area relative to WC is consistent with the hypothesis of increased breeding competition in BC.

Fluctuations in breeding adult abundance were proportionally about the same in BC and WC between the 2 years where data were available. In addition, our data also suggest that sex ratios do not deviate from one-to-one in both streams, and population subdivision within streams was not supported by the analyses with STRUCTURE. By ruling out the effects of these factors, greater variance in individual reproductive success appears to be playing a major role in the reduction of N_e_ relative to N in BC relative to WC. The similar expected heterozygosities and allelic richness in the two streams also suggest that these isolated populations may have had quite similar N_e_ for some time.

We surmise that exacerbated breeding competition in BC probably arises in both sexes. With spawning taking place at fewer sites, the probability of disproportionately high mating success by certain males may increase in BC. Disproportionately more BC than WC females might also be forced to spawn in suboptimal areas owing to higher population densities, and hence might be more likely to lose their entire reproductive output to chance events. Another factor could be nest superimposition, a common phenomenon in salmonid fishes (Essington et al. 1998; Steen and Quinn 1999). Again, with fewer spawning sites, such nest superimposition could be more likely and also result in a greater skew in reproductive success in BC than in WC. Although this hypothesis was not tested, a plausible scenario leading to greater reproductive skew could be that later spawning fish have higher offspring survival, resulting in a lower N_e_/N ratio. The reproductive investment by BC females in more, smaller eggs rather than fewer, larger eggs might then be viewed as a bet-hedging strategy in the face of superimposition, though an equally plausible alternative is that the smaller egg size could very well represent a local adaptation to the abiotic conditions present within the breeding site in BC.

Our conclusions regarding enhanced breeding competition as the primary factor explaining the especially low N_e_/N ratio in BC are necessarily restricted by the lack of data replication over several years in both streams (and/or from additional, adjacent streams). Other factors may also be partially responsible, but to disentangle their relative roles, longer term data would need to be collected on within stream N_e_, N, and age-specific survivorship. For instance, while only half a year separates the average generation time between BC and WC trout, differential age-at-maturity can affect N_e_/N ratios (Nunney and Elam 1994). Shorter generation times (resulting from earlier average age-at-maturity) tend to decrease N_e_/N ratios more than longer generation times, yet the converse arises when lower juvenile survival occurs (Lee et al. 2011).

Cape Race trout populations are also iteroparous (Hutchings 1993), so it cannot be ruled out that fluctuating survival rates across years could increase lifetime variance in reproductive success and reduce N_e_ per generation more in BC than WC, even if survival within each year was random. Indeed, early juvenile survival might fluctuate more interannually in BC than WC given that recruitment occurs at fewer sites and that shallower, faster streams can be more sensitive to environmental changes such as floods or severe winters (Allan and Castillo 2009). However, such an interannual effect on N_e_ is expected to have its strongest effect in semelparous species and would be modulated in iteroparous ones (Waples 2002).

In conclusion, across two adjacent trout populations, we find that little correspondence exists between the amount of habitat available for sustaining later-stage juveniles or adults and the amount of habitat permitting breeding and rearing of early life stages. Moreover, population densities appear to be influenced by specific habitat features that affect individual body size. Combined, these factors likely result in different breeding competition regimes in each population, and concomitant differences in the extent to which adults contribute to their respective gene pools. A similar discordance between juvenile or adult productivity versus breeding habitat availability occurs in other brook trout populations (Ridgway and Blanchfield 1998) and in other taxa, including birds (Newton 1994), mammals (Banks and Dickman 2000), and fishes (Shrimpton and Heath 2003). This suggests that disparate N_e_/N ratios arising through a similar process may be quite common, and perhaps most prominently within species with substantial variation in body size, where the potential for varying influences on density-dependent effects across populations is most likely. The divergent N_e_/N ratios in adjacent populations documented here also provide a cautionary reminder for species conservation programs that genetic and demographic parameters within populations (N_e_, N) may be uncorrelated. Collectively, we anticipate that a fuller consideration of how ecology and evolution interact to generate variability in N_e_/N ratios will be fruitful for evolutionary ecology and conservation biology.

## Figures and Tables

**Figure 2:**
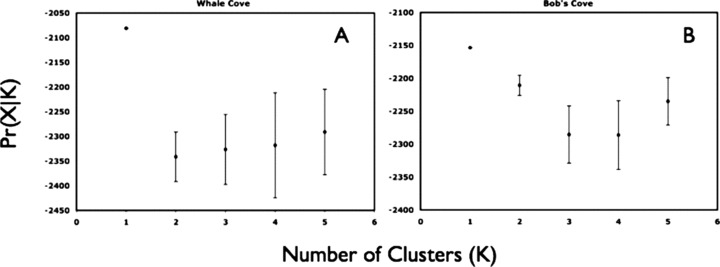
The means and standard deviations of estimated log probabilities for Whale Cove (A) and Bob's Cove (B) rivers after 10 replicate trials in STRUCTURE allowing for admixture and correlated allele frequencies and using a burn-in period of 100,000 replicates and 500,000 MCMC reactions.

## Appendix 1. Detailed Descriptions of Habitat Data Collection Methodology

Habitat data were collected for 21 transects at Bob's Cove river on 6 July 2010, between 7:57 and 14:42, and for 18 transects of Whale Cove river on 10 July 2010, between 9:43 and 18:39. Beginning from the mouth of each stream, transects were spaced approximately 100 m apart and covered the entire length of each stream that was inhabited by brook trout (length of Bob's Cove ∼ 2050 m, and Whale Cove ∼1822 m). Data were collected for 12 different stream characters and measurements at each transect took, on average, 18 min to complete.

### pH

Water pH can have a large effect on numbers of species and individuals in a stream as well as ecosystem processes. At very low pH levels (less than 3), coagulation of mucous on gill surfaces and subsequent anoxia may be the primary cause of acid-induced mortality of fish. At pH 4–5, disturbance of normal ion and acid–base balance is the likely cause of mortality. In one study, fertilization success of Atlantic salmon eggs by large sea-run males and precocious male parr and recruitment of juveniles was found to decline at pH values of less than 5.0 due to decreasing spermatozoan motility; no eggs were fertilized below pH 4.0 (Daye and Glebe 1984). For brook trout, the selection of underground spring areas for spawning reduces the risk that low pH will affect the survival of early life stages, however, the critical time for young brook trout may be when they emerge from the redd. As alevins emerge to begin feeding exogenously, they may be forced to cross a chemical gradient representing a more than 100-fold increase in H^+^. Though brook trout have been found to be very tolerant of low pH (Grande et al. 1978), the shock of crossing from alkaline to very acidic water may cause high mortality (Gunn 1986). We measured pH using a WTW Multiline P4 universal meter. At each transect, the probe was submerged in the stream and held in place until the reading stabilized.

### Mean temperature

Temperature is a critical environmental variable determining the metabolic rates of organisms, as well as their distribution along a river's length. Because species compositions and metabolic rates are temperature dependent, ecosystem processes including leaf breakdown, nutrient uptake, and biological production are also affected (Allan and Castillo 2009). Among fish, temperature is an important factor controlling not only metabolism and growth, but also the timing of spawning and emergence (Brett et al. 1969). Stream temperature at Cape Race was measured in conjunction with pH using the WTW Multiline P4 universal meter pH probe. Stream temperature at each transect was measured mid-channel and away from large objects projecting above the water surface, to avoid elevated readings. The probe was held just below the water surface for the amount of time required for pH readings to stabilize, approximately 10 min.

### Mean velocity

Current velocity influences both channel shape and substrate composition and strongly affects ecological interactions, rates of energy transfer, and resource distribution within the stream environment (Hart and Finelli 1999). Flow conditions are important to ecosystem processes through the delivery of nutrients and gases and removal of wastes, and by influencing which age classes or even species occur at a site. For drift feeding fish such as brook trout, capture rate is a function of visual reaction distance, depth, and velocity; swimming costs also depend on velocity (Hughes and Dill 1990). Velocity in this study was measured using a ball attached to a 1-m string. Holding one end of the string, the ball was released from an upstream direction by one person and allowed to reach the end of the string. A second person recorded the time required for the ball to travel 1 m using a stopwatch. The velocity was calculated as 1 m divided by the time required for the ball to travel the length of the string to obtain a velocity estimate in m/sec. Mean velocity of the transect was calculated as the average of three measurements spaced equally across the width of the stream channel.

### Percent riparian cover

Riparian vegetation can help to moderate stream temperature by reducing the amount of solar radiation reaching the water surface (Beschta et al. 1987). For salmonids, riparian vegetation provides important thermal refuges as well as cover from avian predators. Allochthonous inputs of riparian vegetation also provide important resource subsidies to aquatic environments affecting microbial, invertebrate, as well as fish communities (Allan and Castillo 2009). We visually estimated percent riparian cover as the percentage of the stream channel that was shaded by overhanging streamside vegetation.

### Number of plant species per transect/percent transect with vegetation

As an indirect indicator of stream productivity, we recorded the number of different species of aquatic submerged and partially submerged vegetation present in each transect as well as visually estimated the proportion of substrate covered by aquatic vegetation for each stream transect. The diversity and abundance of aquatic vegetation dictates the diversity and abundance of stream invertebrates that are a primary food source for stream-dwelling salmonids.

### Bank width

Bank width is a character that provides a measure of stream size and therefore the quantity of habitat available for salmonids (Kaufmann 1993). If space is limiting in small habitats, then mortality and reproduction may occur in a density-dependent manner, and individual success will be determined by relative competitive ability. Previous studies have found positive relationships between stream width and trout presence or abundance (Nelson et al. 1992; Clarkson and Wilson 1995; Kruse et al. 1997; Dunham and Rieman 1999). Channel width may also affect in stream temperature regimes, since wider channels will have less riparian shading and more surface area exposed to direct sunlight (Allan and Castillo 2009). We measured bank width as the horizontal distance along a transect, from bank to bank at the existing water surface using a measuring tape.

### Stream length

Stream length is another metric related to stream size and the quantity of habitat that could potentially be used by salmonids. We measured stream length using the path tool of Google Earth; length was measured along the thalweg from the mouth to the end of each stream.

### Mean depth

Stream depth is an important characteristic for stream-dwelling salmonids since water temperature is directly influenced by stream depth; the shallower the stream, the more temperature will fluctuate in accordance with air temperature changes. One manipulative experiment that increased the number of deep pools caused increased abundance of adult trout in six northern Colorado streams (Gowan and Fausch 1996). Deep, low-velocity pool habitats with undercut banks are likely to be especially critical refuges for fish in Cape Race streams since there is a noted absence of riparian shading in many streams; pools also provide critical overwintering habitat. Depth was measured as the mean of five equally spaced points along the transect using a meter stick.

### Drainage area

Drainage area dictates stream size; larger streams can support larger populations, which may be less vulnerable to environmental and demographic stochasticity (Lande 1993), and are more likely to contain the diverse range of habitat types needed by salmonids at different stages of life history. For example, juveniles are typically found in shallow, riffle areas, whereas adult fish prefer large, deep pools (Gibson and Cutting 1993). In one study, watershed area was the one basin-scale habitat attribute found to be useful as a coarse filter for predicting translocation success of cutthroat trout (Harig and Fausch 2002). Large watersheds are also likely to have sufficient input of large woody debris and boulders to create physical structure in pools. Drainage area was measured in this study using MapWindow open source GIS software (http://www.mapwindow.org/downloads/index.php?show_details=1, MapWindow Open Source Team 2008) in conjunction with the MSWAT plug-in (SWAT Development Team 2009). Digital elevation maps (90-m resolution) for Cape Race were obtained at http://srtm.csi.cgiar.org/SELECTION/inputCoord.asp, Jarvis et al. 2008). Once the drainage basins had been delineated using MSWAT, an additional plug-in was used to convert the shape file to a KML-formatted overlay that was then imported into Google Earth (Google 2011). Drainage areas were then calculated using ImageJ (Rasband 2011). Details on data preprocessing, and the use of MSWAT can be found at the WaterBase website: http://www.waterbase.org/ (Briley 2010; Leon 2007, 2010).

### Spawning sites

Amount of spawning habitat is a crucial factor effecting reproductive success in salmonid populations. When spawning habitat is limited relative to the abundance of potential breeders in a population, there is increased competition for spawning territories and mates, as well as increased risk of redd superimposition and embryo mortality due to delayed spawning. Adequate spawning habitat alleviates these issues and allows a larger percentage of potential breeders to contribute to future generations, helping to decrease genetic problems such as inbreeding in small populations (Arden and Kapuscinski 2003). Total number of spawning sites at Bob's Cove and Whale Cove was recorded for each stream in the fall of 2010. Spawning sites were easily recognizable by dense aggregations of sexually mature individuals and by the presence of *Montia fontana*, a herbaceous annual plant that is indicative of groundwater seepage at Cape Race; brook trout are known to preferentially spawn in areas with upwelling groundwater (Witzel and MacCrimmon 1983; Curry and Noakes 1995).

### Stream invertebrates

Invertebrates play an important functional role in stream environments by influencing decomposition, primary production, and nutrient spiraling and are furthermore a primary source of food for salmonids and other fish (Rader 1997). Invertebrate abundance was assessed for three different sites within Bob's Cove and Whale Cove rivers on 22 July 2010, between the hours of 1100 and 1400. Since benthic feeding has been found to be prevalent among YOY age classes (Grant and Noakes 1987), we used a combination of both drift- and kick-sampling methods. Drift nets were placed at three riffle sites within each stream for 30 mins each on 22 July 2010 between the hours of 1100 and 1400. Kick samples were obtained by disturbing the substrate for 1 m immediately upstream of the net opening. Invertebrates in each sample were identified using a dissecting microscope and placed in a vial of 70% alcohol.

## Appendix 2. Subpopulation Analysis Using STRUCTURE

To determine if there was any subpopulation structure within either stream, the Bayesian clustering method available in STRUCTURE (Pritchard et al. 2000) was implemented. For each stream, 10 independent trials under a model of admixture and correlated allele frequencies were performed using *K-*values ranging from one to five. A burn-in period of 100,000 replications followed by 500,000 Monte-Carlo Markov chain (MCMC) reactions was used, and the estimated posterior probabilities of the data Pr(X∣K) (Pritchard et al. 2000) were recorded for each trial. Delta *K* was calculated for *K*= 2 through *K*= 4 following the procedure outlined in Evanno et al. (2005).

The high mean Pr(X∣K) at *K*= 1 (Figure 2a,b) for each population suggests that neither population contains subpopulation structure. This is further supported by the relative lack of any peak Δ*K* (graph not shown) suggesting that each population contains only one cluster.
